# Survival of death certificate initiated registrations: selection bias, incomplete trace-back or higher mortality?

**DOI:** 10.1038/sj.bjc.6603418

**Published:** 2006-11-21

**Authors:** P Silcocks

**Affiliations:** 1 Trent Cancer Registry, 5 Old Fulwood Road, Sheffield S10 3TG, England; 2 Trent Research & Development Support Unit, 14th Floor, Tower Building, University Park, Nottingham NG7 2RD, England

**Keywords:** cancer registration, DCI cases, cancer survival, routine statistics

## Abstract

Cases first notified to a Registry and successfully followed back have an apparently worse prognosis than cases registered in life. A simple approach can be used to assess whether this is due to selection bias, incomplete follow-back or intrinsically higher mortality. For the colorectal, breast and stomach cancers studied and for comparable registries, the main explanations are likely to be selection bias and higher mortality.

Death Certificate Only registrations (DCOs), well known to cancer registries, are registrations for which the death certificate is the only evidence for a diagnosis of cancer. Such cases are a subset of those registrations that are only initiated after death, and may be termed *Death Certificate Initiated* registrations (DCIs), or *Death Certificate Notifications* (DCNs). Most DCIs can, after follow-back, be linked successfully to hospital records to obtain the original date of diagnosis and it is useful to introduce the term *traced DCIs* for these. Death certificate only registrations therefore arise either when attempts to follow-back DCIs fail (because a hospital record cannot be found at all), or when DCIs are successfully linked to hospital records but these records give an alternative diagnosis or contain no supporting evidence for cancer. It has therefore been recommended that the latter category should be specially flagged or even excluded from cancer incidence estimates (Powell, 1991); this is not universal practice, however.

Although the percentage of DCO registrations is an important measure of the quality of registry data (Parkin *et al*, 1994), in many registries the reported percentage of DCOs includes both DCOs and DCIs for which no follow-back attempts have been made. It is important to be aware, therefore, that the reported percentage of DCOs may overestimate the true percentage of DCOs as defined above (% true DCO), depending on how much resource is devoted to follow-back.

Most cancer registrations, however, are made from hospital records (including pathology and other hospital-based sources such as clinical databases) while the patient is still alive. There seems to be no standard term for this broad category and in this paper they are referred to as *Registered In Life* (RIL) registrations.

It is well known in registries that traced DCI cases have worse survival than RIL cases (see Figure 1), and for this there are three possible explanations:

- selection bias, because DCIs are far from being a random sample of cases;
- incomplete follow-back, because the earliest recorded date is derived from a more recent hospital attendance than the actual diagnosis date. This may occur, for example, if the original medical notes are missing, and the diagnosis date is taken from attendance for treatment of recurrent disease;
- intrinsically greater mortality, due to greater age, co-morbidity, or more advanced or more aggressive disease.

This study aimed to quantify these possibilities by taking RIL cases from a registry database and modifying the records to see how big the changes would have to be to produce a survival curve resembling that of actual traced DCIs.

## METHODS

All colorectal cancer registrations for 2000 in the Trent Region were extracted from the Registry database in January 2005 and classed as DCO, Traced DCI or RIL.

**Figure 1:**
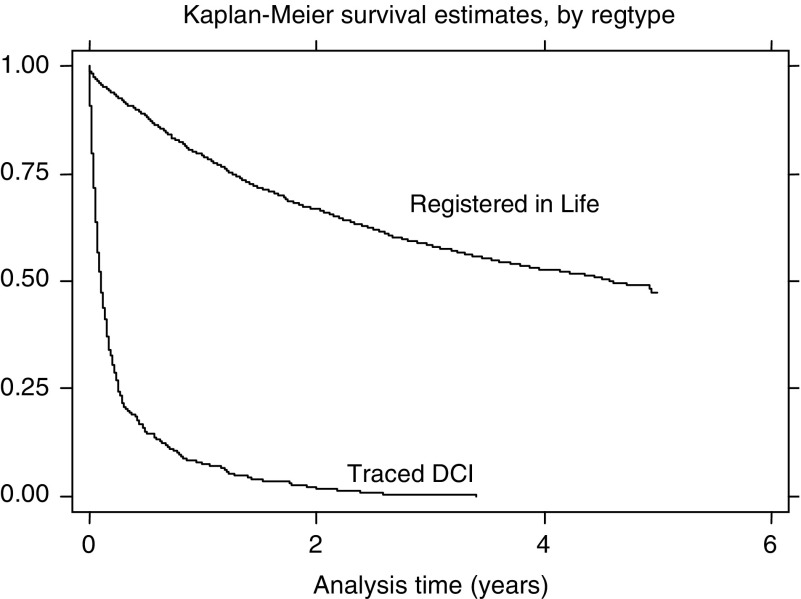
Survival of Registered in Life and traced DCI cases (colorectal cancer).

The survival of traced DCI cases was compared with that of RIL cases by Kaplan–Meier survival plots (Figure 1). It was assumed that the separate hazard ratios of selection bias, incomplete follow-back and mortality would be multiplicative, their product being the hazard ratio observed for traced DCI cases relative to RIL cases, denoted by *R*_*DCI*_, which is the value to be accounted for in possibilities (a) to (c). This model supposes that if *S*_*t*_ is the survival of cases registered in life at time t after diagnosis, then the survival of DCI cases is given by 
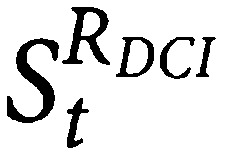
 with *R*_*DCI*_ being given by: 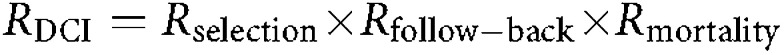 where *R*_DCI_ is observed directly, *R*_selection_ is estimated from dead RIL cases, *R*_follow-back_ is estimated using ‘reasonable’ assumptions and *R*_mortality_ is what remains.

To answer possibility (a) the RIL cases were duplicated and then split into two equal groups. One group was labelled as ‘pseudo-DCIs’ and for this group only cases that had died were retained (because DCIs are, by definition dead). Comparison of the survival (as before) of these pseudo-DCIs with the remaining RIL cases indicated the contribution of selection bias alone through hazard ratio *R*_selection_. The reduced hazard ratio due only to incomplete follow-back and mortality, *R*_DCI_reduced_=*R*_follow-back_ × *R*_mortality_, was estimated as: *R*_DCI_/*R*_selection_.

**Figure 2:**
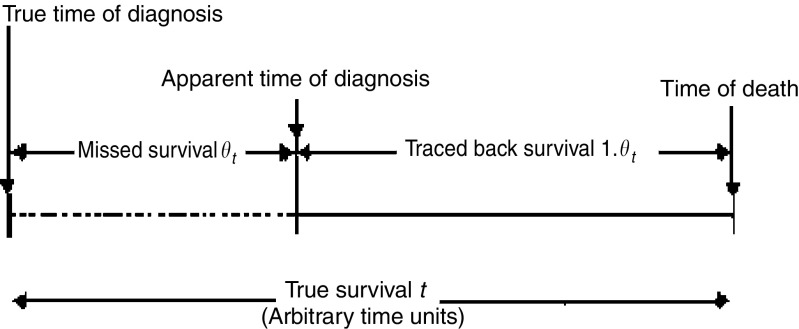
Imputing missed survival.

To assess possibility (b) – the contribution of incomplete follow-back – it was assumed that for any individual traced DCI case, a maximum fraction of the true survival time *t* will be missed (see Figure 2). For simplicity, assuming that the missing increment has a uniform distribution on the interval [0, *θt*] then the *average* amount of time missed is ½*θt*. As the survival time actually observed due to follow-back, *t*_traced_ must then be (1−*θ*)*t*, the missing increment will have a uniform distribution on the range (0, *θ*.*t*_traced_/(1–*θ*)) by substituting *t*=*t*_traced_/(1−*θ*); the *average* amount of time missed will be half of this, that is ½*θt*_traced_/(1–*θ*). The survival time based on follow-back can then be corrected for an assumed fraction missing by adding this increment. An alternative approach was also tried, in which the missed increment has a negative exponential distribution with the same mean, which might be more realistic as it does not constrain the maximum amount of time that could be missed. The choice of model does not affect the value for *R*_selection_ or of course the observed value *R*_DCI_.

As correcting for incomplete follow-back *increases* the survival time for pseudo-DCIs, the effect is to *reduce* the hazard ratio relative to RIL cases, so the corrected hazard ratio then observed *R*_corrected_ is actually (*1/R*_follow-back_) × *R*_selection_. We already know *R*_selection_ so we can estimate *R*_follow-back_ as *R*_selection_/*R*_corrected_. Note that because these are *pseudo*-DCIs there is no effect of intrinsically higher mortality.

The hazard ratio *R*_follow-back_ was then estimated for different values of *θ*, and the critical value at which *R*_follow-back_ equalled the value unaccounted for by selection bias was found by cubic interpolation.

Finally to address possibility (c), for each value of *θ* the hazard ratio among real traced DCIs due to intrinsically higher mortality was estimated by *R*_mortality_=*R*_DCI_ */(R*_selection_ × *R*_traceback_).

Similar analyses were performed for stomach and breast cancers representing tumours with worse and better survival respectively (these had not been subject to extensive follow-back activity).

Calculations were performed in Stata version 9.

## RESULTS

Of 2896 colorectal cases, 16.9% were DCIs, 4.4% were DCOs and the remainder were registered in life. A cumulative hazards plot (Collett, 1994) showed that the proportional hazards assumption was reasonable for comparing traced DCIs and RIL cases at least for 5 years survival; the hazard ratio of traced DCIs to ‘known’ cases was 12.32 (Figure 1).

Table 1 displays the results for different assumed proportions missing. It is clear that incomplete follow-back (on top of selection bias) cannot account for the poorer survival of DCIs unless the maximum proportion missing is more than 80% (40% missed on average). For ‘plausible’ maximum proportions lost in the region of 10–20% *R*_mortality_ was at least twice that of *R*_follow-back_, although still smaller than *R*_selection_. Table 1 also shows that the results of the alternative exponential model for the missed survival time were similar.

The supplementary analyses for stomach and breast cancer are shown in Table 2, using only results from the uniform distribution model. The general conclusions are similar, but selection bias was relatively less important for stomach cancer while for breast cancer the opposite applied. This may be because selection on the fact of death cannot make much difference to apparent survival for cancers with a very poor prognosis.

## DISCUSSION

The worse survival of DCI cases relative to RIL cases was partly, but by no means entirely, reproduced by selection bias. To obtain a large contribution due to incomplete follow-back required an implausibly high proportion of missed survival time, and with more plausible values the main factors were selection bias and intrinsically higher mortality. This result may be helpful when, for example, building simulation models of cancer registration in order to decide whether the effect of incomplete trace-back may reasonably be ignored.

Limitations of this study are, firstly, the extent to which a proportional hazards model applies for each component. This proportionality assumption does not have to be true, as long as it is a reasonable approximation – but of course, as we are dealing with unknowns the approach also has the advantage of simplicity. In any case the results should be viewed as indicative rather than exact. Secondly, the way the missed survival time has been estimated automatically may give a greater increment to cases with longer traced survival, although formulating the missed time as a fraction of the true value seemed a natural approach. An attempt to mimic missed increments inversely related to the followed-back survival resulted in markedly crossed survival curves with a strange sigmoid survival for pseudo-DCIs, so this seems unlikely to be realistic. Thirdly, the results strictly apply only to colorectal, breast and stomach cancer, and to registries with similar practices to Trent Cancer Registry; nevertheless the approach described could easily be adopted generally.

The key lesson of this paper is that as incomplete follow-back of survival is unlikely to miss much survival time, registry resources devoted to follow-back should be directed towards linking a higher proportion of DCI patients with hospital records, rather than scrupulously checking the completeness of each patient's history. There are also opportunities for further research, for example: validation by intensive efforts to obtain actual diagnosis dates for DCIs; similar studies of other tumour sites or in registries with different working practices; and the use of different models for the missed survival.

## Figures and Tables

**Table 1:** Breakdown of hazard ratio components assuming multiplicative hazards (colorectal cancer)

**Hazard ratio to be accounted for: *R*_DCI_**				**12.32**		
**Due to selection bias *R*_selection_**				**3.80**		
**Unaccounted for by selection bias**				**3.25**		
**Uniform distribution**				**Exponential distribution**		
**Max prop missed**	**Average prop missed (%)**	***R*_traceback_**	***R*_mortality_**	**Average prop missed (%)**	***R*_traceback_**	***R*_mortality_**
0.10	5	1.06	3.07	5	1.06	3.05
0.15	7.5	1.10	2.94	7.5	1.11	2.92
0.20	10	1.15	2.82	10	1.15	2.82
0.30	15	1.27	2.55	15	1.25	2.59
0.40	20	1.42	2.28	20	1.38	2.35
0.50	25	1.60	2.03	25	1.54	2.10
0.60	30	1.86	1.75	30	1.86	1.82
0.70	35	2.24	1.45	35	2.24	1.54
0.80	40	2.96	1.10	40	2.65	1.23
0.82*	41*	3.25	1.00	41*	3.25	1.00

* Value accounting for all excess apart from selection bias.

**Table 2:** Breakdown of hazard ratio components assuming multiplicative hazards (stomach and breast cancers)

	**Uniform distribution**		
**‘Max’ prop missed**	**Average prop missed (%)**	***R*_traceback_**	***R*_mortality_**
(a) Stomach cancer			
Hazard ratio to be accounted for *R*_DCI_		5.39	
Due to selection bias *R*_selection_		1.64	
Unaccounted for by selection bias		3.28	
0.10	5	1.04	3.15
0.15	7.5	1.07	3.08
0.20	10	1.09	3.01
0.30	15	1.16	2.82
0.40	20	1.26	2.60
0.50	25	1.39	2.36
0.60	30	1.60	2.09
0.70	35	1.85	1.77
0.80	40	2.35	1.40
0.88*	44*	3.28	1.00
			
(b) Breast cancer			
Hazard ratio to be accounted for *R*_DCI_		21.70	
Due to selection bias *R*_selection_		10.12	
Unaccounted for by selection bias		2.14	
0.10	5	1.09	1.96
0.15	7.5	1.15	1.86
0.20	10	1.24	1.74
0.30	15	1.48	1.45
0.40	20	1.74	1.23
0.50*	25*	2.14	1.00

* Value accounting for all excess apart from selection bias.
